# The effect of general anesthesia and conscious sedation in endovascular thrombectomy for acute ischemic stroke: an updated meta-analysis of randomized controlled trials and trial sequential analysis

**DOI:** 10.3389/fneur.2023.1291211

**Published:** 2023-12-08

**Authors:** Zhi Peng, Wenmiao Luo, Zhengcun Yan, Hengzhu Zhang

**Affiliations:** ^1^Department of Neurosurgery, Clinical Medical College of Yangzhou University, Yangzhou, China; ^2^Department of Neurosurgery, Affiliated Renhe Hospital of China Three Gorges University, Yichang, China; ^3^Department of Neurosurgery, Xiamen Susong Hospital, Xiamen, China

**Keywords:** EVT, AIS, general anesthesia, conscious sedation, meta-analysis

## Abstract

**Objectives:**

General anesthesia (GA) and conscious sedation (CS) are common methods for endovascular thrombectomy (EVT) in acute ischemic stroke (AIS). However, the risks and benefits of each strategy are unclear. This study aimed to summarize the latest RCTs and compare the postoperative effects of the two methods on EVT patients.

**Materials and methods:**

We systematically searched the database for GA and CS in AIS patients during EVT. The retrieval time was from the creation of the database until March 2023. The quality of the studies was evaluated using the Cochrane risk of bias tool. Random-effects or fixed-effects meta-analyses were used to assess all outcomes.

**Results:**

We preliminarily identified 304 studies, of which 8 were included. Based on the pooled estimates, there were no significant differences between the GA group and the CS group in terms of good functional outcomes (mRS0-2) and mortality rate at 3 months (RR = 1.09, 95% CI: 0.95–1.24, *p* = 0.23) (RR = 0.95, 95% CI: 0.75–1.22, *p* = 0.70) as well as in NHISS at 24 h after treatment (SMD = −0.01, 95% CI: −0.13 to 0.11, *p* = 0.89). However, the GA group had better outcomes in terms of achieving successful recanalization of the blood vessel (RR = 1.13, 95% CI: 1.07–1.19, *p* < 0.0001). The RR value for the risk of hypotension was 1.87 (95% CI: 1.42–2.47, *p* < 0.00001); for pneumonia, RR was 1.43 (95% CI: 1.07–1.90, *p* = 0.01); and for symptomatic intracerebral hemorrhage, RR was 0.94 (95% CI: 0.74–1.26, *p* = 0.68). The pooled RR value for complications after intervention was 1.03 (95% CI, 0.87–1.22, *p* = 0.76).

**Conclusion:**

In patients undergoing EVT for AIS, GA, and CS are associated with similar rates of functional independence. Further trials of a larger scale are needed to confirm these findings.

## 1 Introduction

For centuries, doctors have conducted numerous drug and surgical trials and innovations to seek effective treatments for stroke patients (1). For example, antiplatelet drugs and intravenous thrombolytics have gradually been applied (2). At the same time, the updating of surgical-assisted technologies has also promoted the development of interventional neuroradiology, providing a method of delivering thrombolytic agents to the occlusive site, i.e., endovascular thrombectomy (EVT) for stroke (3). In the past few years, several influential RCTs have demonstrated the efficacy and safety of EVT for AIS (4–6). However, EVT treatment has many factors that affect the prognosis of patients, such as time, the speed of treatment, and hemodynamic status, and some scholars further consider the impact of anesthesia methods on the overall prognosis of AIS patients. Some doctors prefer intubated GA, believing that GA may be associated with spasms, anxiety, excitement, and movement, and reduces inhalation risk. Others tend to use CS to save time, cause less hemodynamic instability, and reduce complications associated with mechanical ventilation (7–14). There is uncertainty about the effect of GA and CS on functional outcomes, and due to the lack of evidence, guidelines do not provide formal recommendations. Both methods have advantages and disadvantages, and the choice of the best anesthesia method for EVT treatment is still controversial.

Summarizing the randomized trials comparing GA and CS for the prognosis of AIS treated with EVT since 2015, numerous scholars have conducted a series of meta-analyses (15–18). Previously, some scholars found that patients receiving GA treatment had certain advantages in functional outcomes compared to the CS group. However, the meta-analysis results of more and more RCTs show that the difference in postoperative functional independence between the GA group and the CS group is gradually narrowing. For example, the meta-analyses of Bai in 2021 and Lee in 2022 found that the *p*-value of the summarized mRS scores of the two groups of patients showed a trend of close to no difference at 3 months after treatment (17, 18). Combining the latest RCTs by Maurice and Liang in the past 2 years (13, 14), this study summarizes the relevant RCTs in order to provide new clinical evidence for the choice of anesthesia method for AIS patients receiving EVT.

## 2 Methods

The preferred reporting items for systematic reviews and meta-analyses (PRISMA) declaration is adhered to by the study procedure (19). On international platforms for systematic reviews and meta-analyses, such as the international registry of systematic review protocols, the final protocol has been registered (PROSPERO: CRD42023423369). Given its nature, patient permission and ethical approval are not necessary for this study.

### 2.1 Eligibility criteria and search strategy

We conducted a systematic search based on the PICOS principles: P, acute ischemic stroke patients; I, general anesthesia; C, conscious sedation; O, mortality rate; S, randomized clinical trials and sequential analysis. Our primary interest lies in the outcome measure of mRS score, with secondary outcomes including mortality rate, NIHSS score, low blood pressure, pneumonia, and SICH. The five electronic databases used in the search technique were PubMed, Cochrane, Embase, Scopus, and Ovid. Studies conducted in languages other than English, letters, comments, and unpublished data were disregarded. Only the most recent publication was taken into account when more than one eligible publication covered the same patient. The references to the articles in other pertinent periodicals were examined, and duplicate articles were eliminated. The retrieval time was from the creation of the database until March 2023. Stroke, anesthesia, endovascular treatment, and other variations of these phrases were utilized as extended search terms. The author’s contributions go into great depth on the whole search process.

### 2.2 Publication selection

The titles and abstracts of the articles received were examined separately by two writers (WL and ZP). The article was included for full-text review if the reviewer thought it related to the research question. The same two writers individually assessed each full-text article. The senior research fellow (HZ) settled any disagreements between the two reviewers. For articles that matched the qualifying requirements, data were extracted.

### 2.3 Data extraction

Two reviewers, WL and ZP, independently extracted the data using the study’s pre-built data extraction form. HZ, a third reviewer, was consulted to resolve disagreements in such instances. When possible, assessors made an effort to get in touch with the study’s lead author to collect any missing information and have it verified.

### 2.4 Risk of bias evaluation

Along with other research data, the name of the trial, publication year, nation, data source, inclusion and exclusion criteria, results, and sample size for each group were all retrieved. The baseline patients’ gender, admission NIHSS score (ranging from 0 to 42, with higher scores indicating more severe functional impairment), and admission mRS score (ranging from 0 to 6, with lower values suggesting independent living) were also retrieved.

### 2.5 Quality assessment and bias

The studies examined the risk of deviation and application using the Revman5.4 program and the Cochrane Collaboration’s method for measuring bias risk. Depending on specified standards, the risk of bias was graded as low, uncertain, or high. If a study had two or more high-risk elements, we classed it as having a medium risk of bias. Studies with a high risk of bias were defined as having more than four high-risk components, whereas studies with a low risk of bias were defined as having 0 to 2 high-risk components (20).

In systematic reviews, meta-analyses that compile data from several trials frequently serve as the primary source of evidence. Re-analyzing the data with fresh experiment results, however, can result in an increase in random error. The TSA viewer version 0.9.5.10 Beta was used to perform TSA in order to reduce the possibility of false-positive results as a result of multiple tests and sparse data (21). By analyzing the relationship between the cumulative *Z*-curve and the TSA bounds, the robustness of the cumulative evidence previously presented was put to the test. The trial sequential monitoring boundaries and the needed information size (RIS) were computed previously. A two-sided test was performed to determine the relative risk of the binary outcomes; type I error, power, and relative risk reduction were set at 5%, 80%, and 20%, respectively (22).

### 2.6 Data analysis

Based on original data, each outcome measure of patients receiving GA and CS was evaluated in each RCT. The main result was satisfactory functional status at 90 days (measured as mRS score ≤2). Short-term postoperative neurological function (NIHSS score), 90 days mortality, effective recanalization, vascular complications, pneumonia, SICH, and intervention-associated complications were all considered secondary outcomes. When comparing GA to CS, the connection between the two variables was evaluated using RR and 95% CIs. If significant heterogeneity exists (*p* < 0.1 or *I*^2^ > 50%), it is recommended by Cochrane reviews to choose the random-effects model; otherwise, the fixed-effect model is employed. For cross-trial synthesis, random-effects or fixed-effects meta-analysis models were used, and the equivalent *Z*-test was used to assess the statistical significance of the combined RRs and 95% CIs. Analyzing continuous outcomes that were given as mean and standard deviation required the use of standardized mean difference (SMD) and 95% confidence intervals (CIs). We utilized the R software to conduct further meta-regression on the included literature. Based on the information available from the included studies, we selected three groups of factors: (1) study location, categorized as either Europe or China; (2) sample size, distinguishing between trial and control groups with a population size greater than or less than 100; (3) stroke type, categorized as either anterior circulation or posterior circulation. The output results are documented in the article and Supplementary material. Statistical computations were performed using the Cochrane Collaboration’s Review Manager software and R software (23, 24).

## 3 Results

### 3.1 Study selection and characteristics

**Figure 1:**
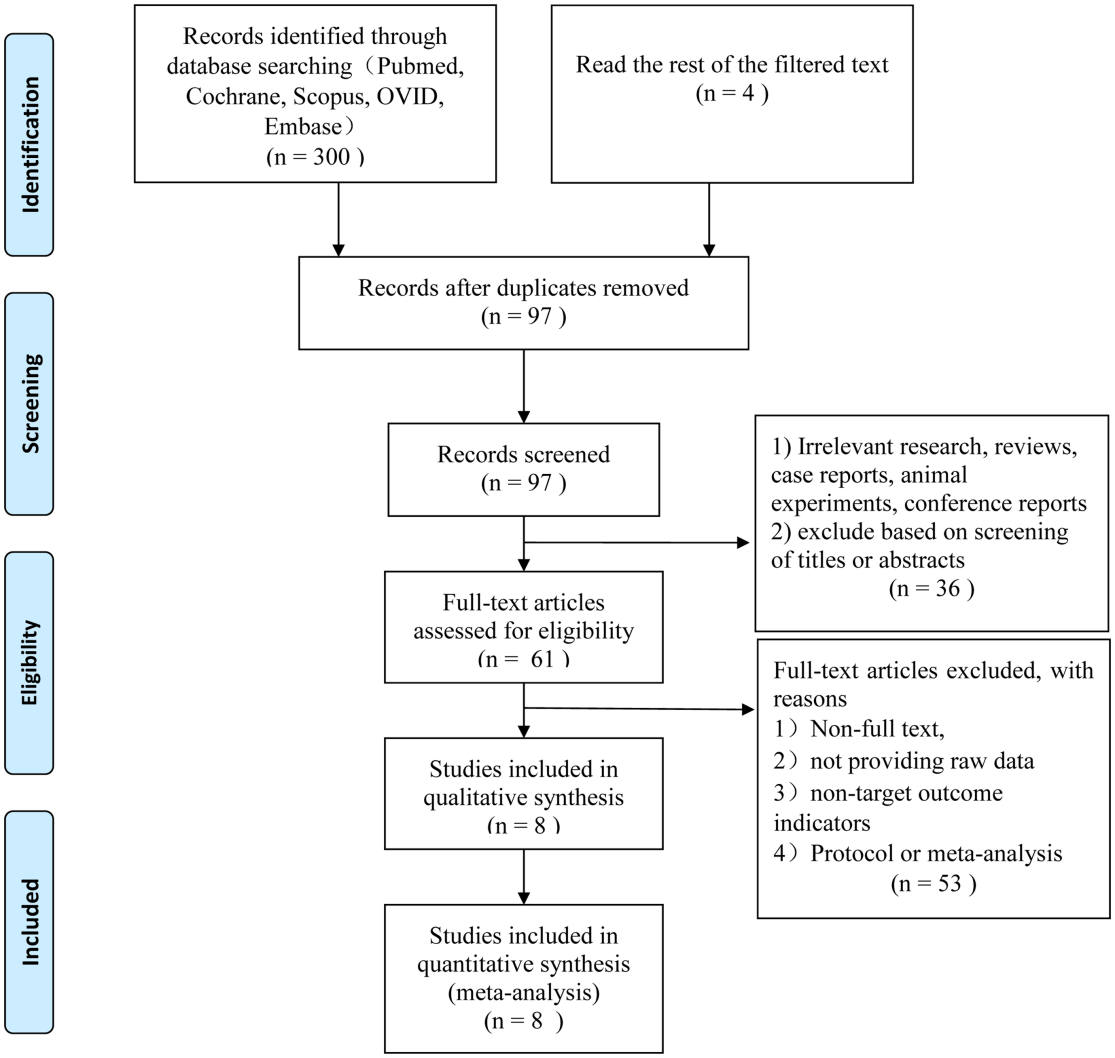
Flow diagram (selection strategy) of included studies.

The search method described above resulted in the first identification of 304 relevant studies. After removing 207 of the duplicates, 97 studies were left for additional evaluation. Thirty-six articles that did not fit the inclusion requirements were disqualified after titles and abstracts were examined. The final eight English language articles were chosen after 53 more studies were eliminated based on exclusion criteria (Figure 1).

The traits of the patients and trials that were considered are shown in Table 1. Five research were done in Europe, and three studies were conducted in China. All studies were randomized controlled trials published after 2015. All included patients’ ischemic stroke locations were confirmed by CT/MRI scans to be within the pre-circulation distribution range. One publication (13) covered acute posterior circulation cerebral infarction, whereas the other seven publications (7–12, 14) focused on acute anterior circulation cerebral infarction. The maximum follow-up time for each of the included randomized controlled studies was 90 days following surgery. The primary outcome was an mRS of 0–2 at 90 days, while secondary outcomes were the mortality rate at 90 days, the rate of postoperative reperfusion, the incidence of postoperative complications, etc. Two studies (7, 9) reported that patients in the GA group had better functional independence at 90 days (an mRS of 0–2) than those in the CS group, while six RCTs (8, 10–14) reported no significant difference. The influence of the two anesthetic procedures on the outcome of EVT therapy for AIS was further examined using the summarized indicators.

**Table 1:** Characteristics of the 8 articles included in the meta-analysis.

Study	2016 SIESTA		2017 ANSTROKE		2018 GOLIATH		2018 Sun		2020 Ren		2022 Liang^*^		2022 Maurice		2023 Chabanne	
Country	Germany		Sweden		Denmark		China		China		China		France		France	
	GA	CS	GA	CS	GA	CS	GA	CS	GA	CS	GA	CS	GA	CS	GA	CS
*n*	73	77	45	45	65	63	20	20	48	42	43	31	169	176	135	138
Age (mean)	71.8	71.2	73	72	71	71.8	67	60	69.21	69.19	64	60	70.8	72.6	72	71.3
Female	25	35	19	22	29	33	7	7	26	24	10	6	80	77	70	72
CS to GA^*^	NA	7	NA	7	NA	4	NA	4	NA	4	NA	13	NA	8	NA	15
Pre-mRS0-2^*^	64	71	44	44	63	63	20	20	48	42	43	44	NA	NA	NA	NA
ASPECTS^*^	8 (7–9)	8 (6.25–9)	10 (8–10)	10 (9–10)	NA	NA	NA	NA	9 (8–10)	9 (8–10.25)	NA	NA	NA	NA	8 (7–9)	8 (7–9)
*Type of EVT*																
Stent retriever	60	66	13	20	14	12	2	3	NA	NA	NA	NA	NA	NA	NA	NA
Direct aspiration	6	4	31	23	25	24	8	9	NA	NA	NA	NA	NA	NA	NA	NA
Both	16	12	12	10	11	10	10	8	NA	NA	NA	NA	NA	NA	NA	NA
IV thrombolysis	20	23	33	36	50	46	9	11	37	34	7	5	111	114	62	70
Onset-to-door time(min)	145.0 (83.8)	118.1 (61.5)	NA	NA	159 (122–230)	145 (113–231)	307 (271–347)	286 (245–333)	262.86 (62.29)	247.38 (33.19)	210 (90–390)	300 (151–450)	89 (57)	88 (53)	NA	NA
*Outcomes*																
mRS0-2 at 90 days	27	14	19	18	44	32	11	10	24	21	21	19	66	63	45	54
Mortality at 90 days	18	19	6	11	5	8	0	0	9	9	10	5	31	28	25	23
Recanalization	65	62	41	40	50	38	19	13	42	36	41	24	144	131	115	107
Po-NHISS after 24 h	13.6 (11.1)	13.6 (9.0)	8 (3–15)	9 (2–15)	6 (3–14)	10 (2–19)	12.4 (5.1)	12.8 (7.3)	NA	NA	NA	NA	11 (9)	11 (7)	9 (3–19)	8 (3–17)
Hypotension risk	NA	NA	41	26	57	22	13	6	28	22	23	3	NA	NA	118	62
Pneumonia	10	3	6	7	NA	NA	10	6	10	2	28	12	NA	NA	26	28
SICH^*^	1	2	0	3	2	1	0	2	9	7	3	1	37	42	21	20
Interventional complications	2	2	11	6	NA	NA	11	14	9	8	34	15	44	55	57	58

Data: mean or median CS to GA, Conversion of conscious sedation to general anesthesia; Pre-mRS0-2, number of mRS0-2 prior to EVT treatment; ASPECTS, Alberta Stroke Program Early CT (computed tomography) Score; SICH, Symptomatic intracranial hemorrhage; 2022 Liang, Liang’s article was the only one of the included articles that reported posterior circulation ischemic stroke. In protocol intention-to-treat of the literature, 43 patients were in the GA group and 44 patients were in the CS group. In the per-protocol group, 43 patients were in the GA group and 31 in the CS group. This meta-analysis included the data of per-protocol group, and the data included in intention-to-treat were analyzed again, and there was no difference in outcome.

### 3.2 Study quality

**Figure 2:**
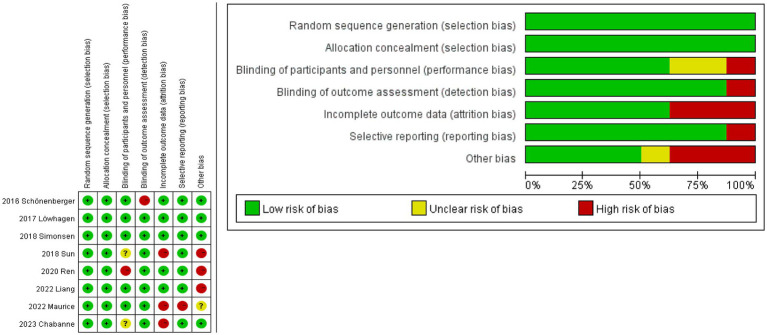
Risk of bias graph. The studies assessed the risk of deviation and its application using the Revman 5.4 software and the Cochrane Collaboration’s bias risk measurement method (red indicates high risk, yellow represents unclear risk, and green signifies low risk). Low deviation (0–2 indicates high risk), moderate deviation (2–4 indicates high risk), and high deviation (more than 4 indicates high risk).

Using the Revman 5.4 software and the bias risk measuring technique created by the Cochrane Collaboration, and bias risk and applicability were investigated in eight studies (Figure 2). Each study gave a thorough explanation of its goals, demographic makeup, methods, and conclusions. The accompanying literature was of a high caliber overall.

### 3.3 Meta-analysis

#### 3.3.1 Primary outcome

**Figure 3:**
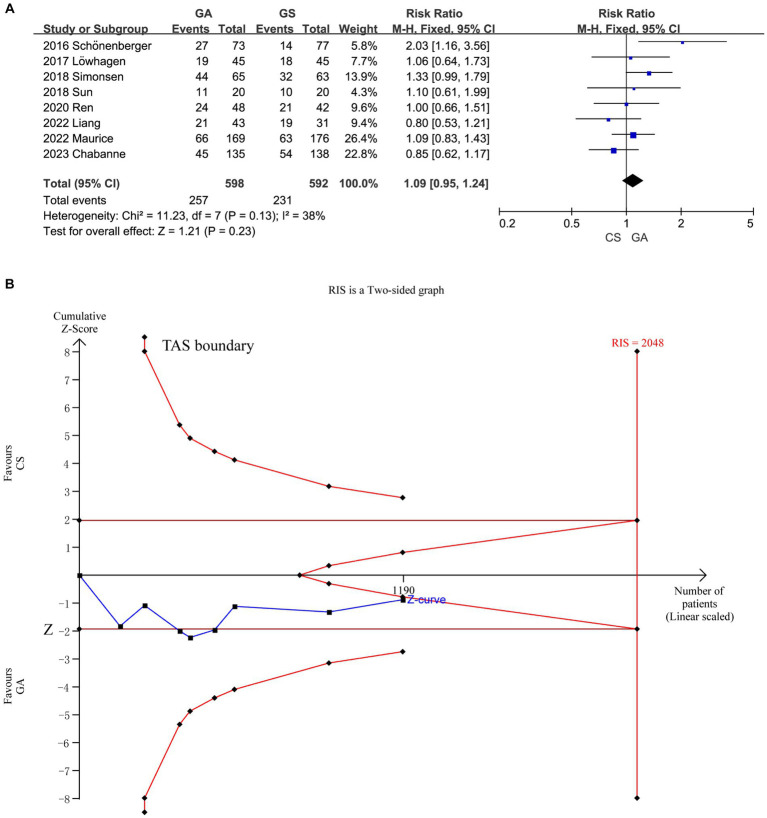
**(A)** Forest plot of functional independence (mRS of 0–2) at 90 days for GA and CS groups. **(B)** Trial sequential analysis of risk of functional independence. The meta-analysis yielded a *Z*-value greater than 1.96, then there was a significant difference between the two interventions studied. RIS refers to the number of cases required for meta-analysis to obtain statistically significant differences. TSA forms a boundary value curve by correcting random error, that is, TSA boundary. Failure to cross the TSA threshold indicates a potentially questionable outcome.

Functional independence after 90 days did not significantly vary between the GA and CS groups (7–14) according to the pooled analysis (*n* = 598 vs. 592) (RR = 1.09, 95% CI: 0.95–1.24, *p* = 0.23). A fixed-effect model was used because of the low heterogeneity (*I*^2^ < 50%) (Figure 3A). Further research is necessary to confirm the possibility that there is no statistically significant difference in treatment effect between the GA and CS groups as the cumulative *Z*-curve on TSA with the required information size (RIS) did not cross (Figure 3B).

#### 3.3.2 Secondary outcome

**Figure 4:**
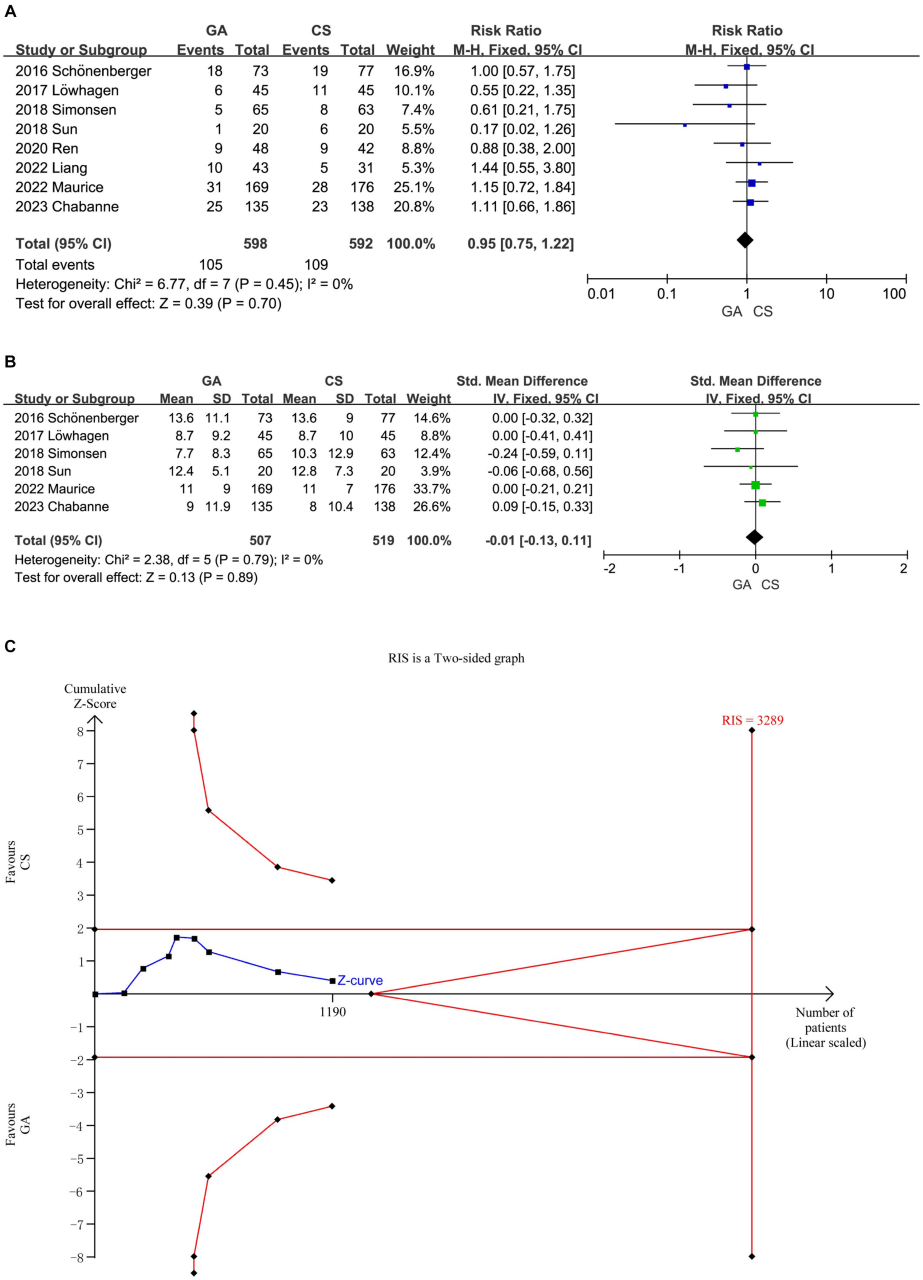
**(A)** Forest plot of mortality at 90 days for GA and CS groups. **(B)** Forest plot of function evaluation (NIHSS score) after 24 h for GA and CS groups. **(C)** Trial sequential analysis of risk of mortality.

The meta-analysis of all (7–14) included literature revealed no significant difference in 90 days mortality rate between the GA group and the CS group (*n* = 598 vs. 592) (RR = 0.95, 95% CI: 0.75–1.22, *p* = 0.70) (Figure 4A), and six studies (7–10, 12, 14) showed no significant difference in NIHSS score between the two groups 24 h after EVT intervention (*n* = 507 vs. 519) (SMD = −0.01, 95% CI: −0.13 to 0.11, *p* = 0.89) (Figure 4B). Low heterogeneity (*I*^2^ < 50%) necessitated the use of a fixed-effect model. More trials (90 days mortality) are required to confirm this according to the cumulative *Z*-curve on the TSA and the non-crossing of the RIS (Figure 4C). The TSA for the difference in NIHSS score was disregarded since there was no enough data to build the TSA border.

**Figure 5:**
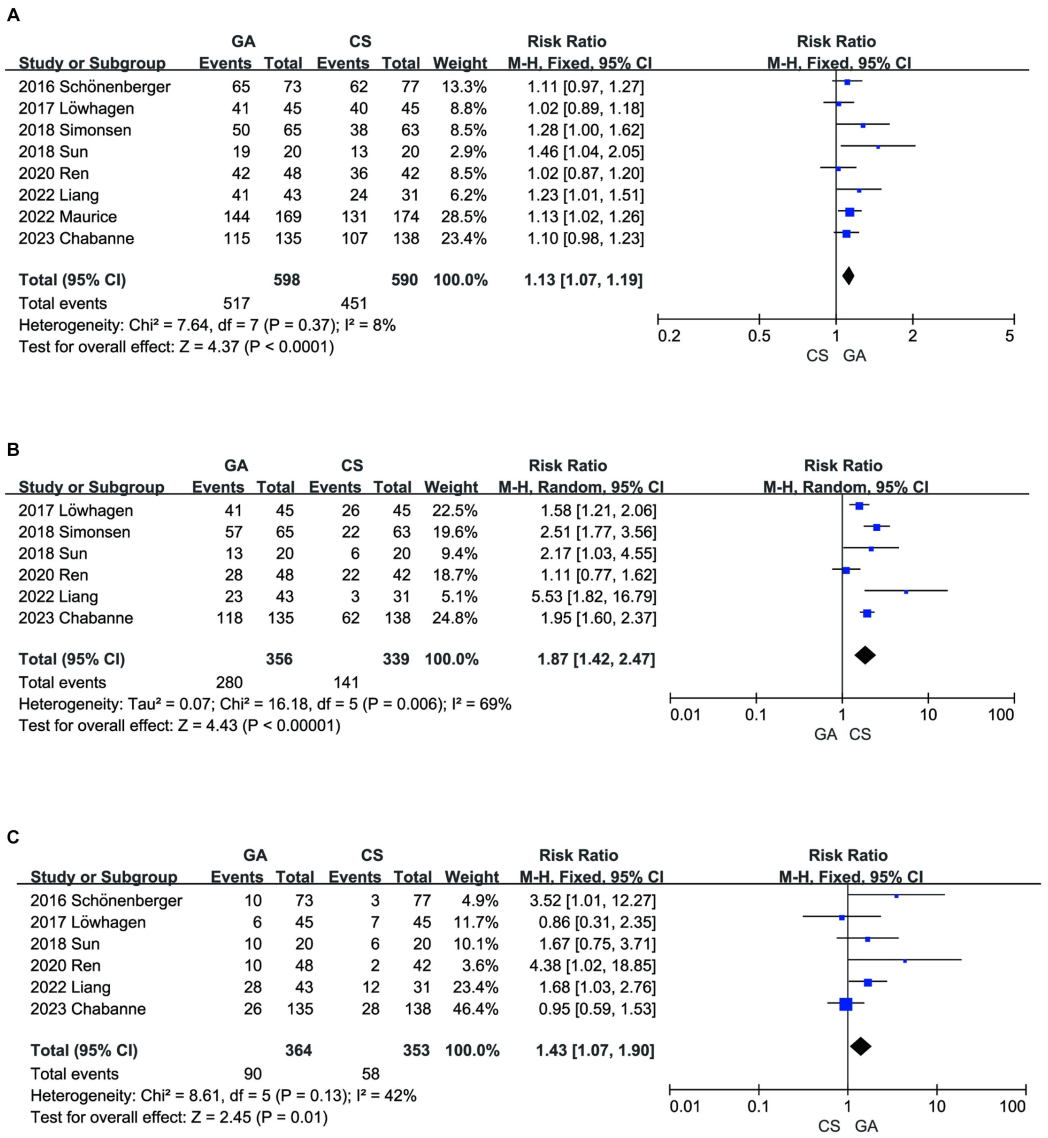
**(A)** Forest plot of recanalization for GA and CS groups. **(B)** Forest plot of hypotension for GA and CS groups. **(C)** Forest plot of pneumonia for GA and CS groups.

Incorporating all the literature (7–14), a meta-analysis demonstrated that compared with the CS groups, the GA groups had a higher recanalization rate after EVT (*n* = 598 vs. 590) (RR = 1.13, 95% CI: 1.07–1.19, *p* < 0.0001) (Figure 5A). However, the literature summarized in this analysis reported a higher risk of hypotension (8–11, 13, 14) (*n* = 356 vs. 339) (RR = 1.87, 95% CI: 1.42–2.47, *p* < 0.00001) (Figure 5B) and pneumonia (7, 8, 10, 11, 13, 14) (*n* = 364 vs. 353) (RR = 1.43, 95% CI: 1.07–1.90, *p* = 0.01) (Figure 5C) in the GA groups compared to the CS groups.

**Figure 6:**
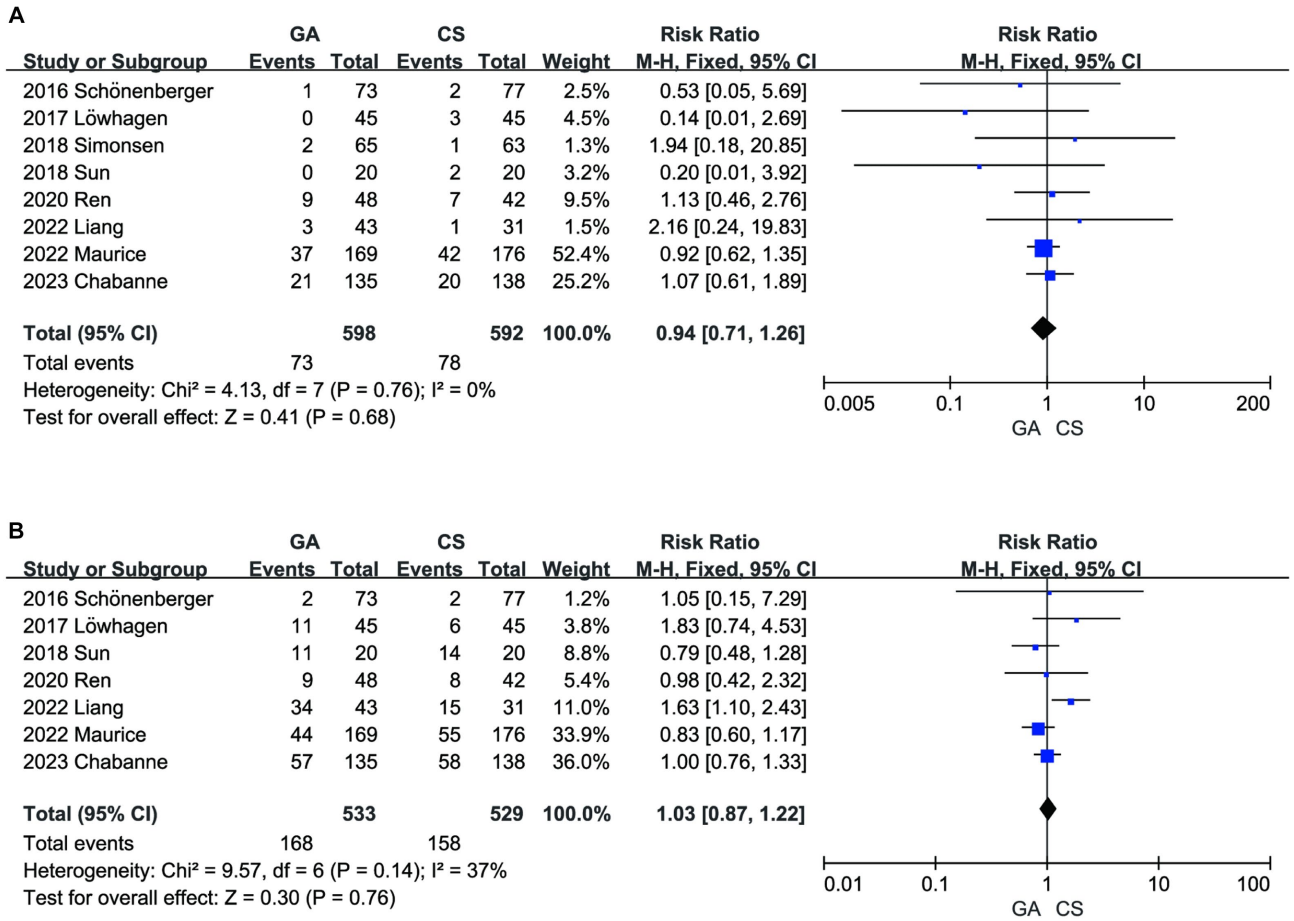
**(A)** Forest plot of SICH for GA and CS groups. **(B)** Forest plot of intervention-associated complications for GA and CS groups.

**Figure 7:**
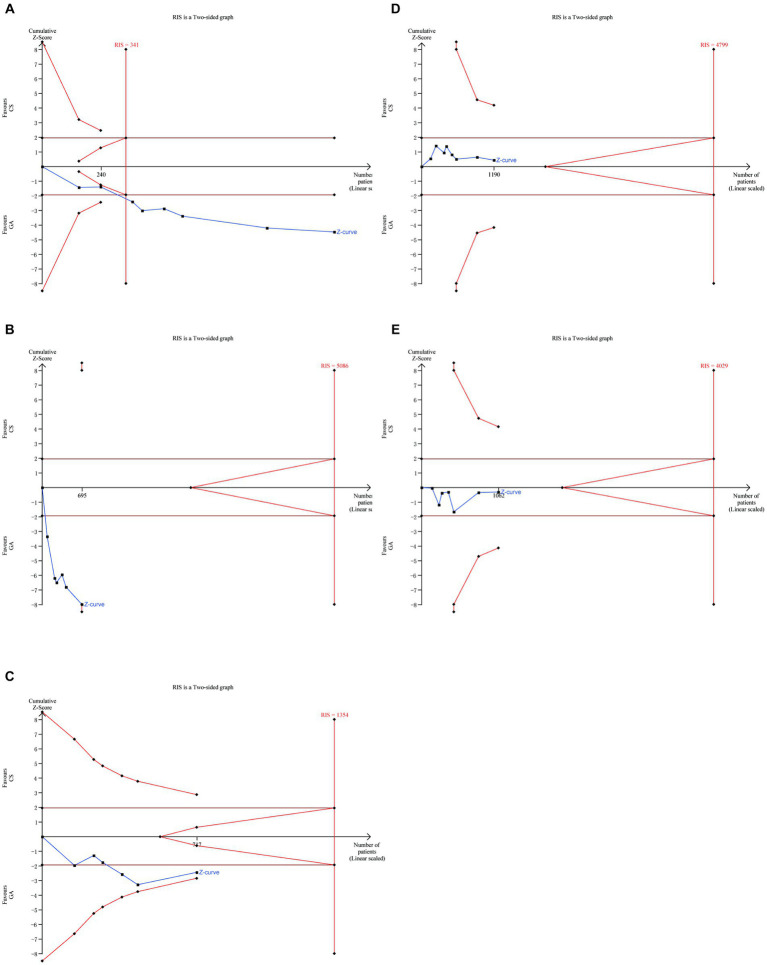
Trial sequential analysis for **(A)** recanalization group, **(B)** hypotension group, **(C)** pneumonia group, **(D)** SICH group, **(E)** intervention-associated complications group.

There was no significant difference between the two groups in terms of SICH (7–14) (*n* = 598 vs. 592) (RR = 0.94, 95% CI: 0.74–1.26, *p* = 0.68) (Figure 6A) and other intervention-associated complications (*n* = 533 vs. 529) (RR = 1.03, 95% CI: 0.87–1.22, *p* = 0.76) (Figure 6B) (7, 8, 10–14). Only the hypotension risk group showed high heterogeneity using a random effects model and the remaining outcomes using a fixed effects model. The cumulative *Z*-curve of the reperfusion rate group in TSA crossed the boundary of the trial sequential monitoring, indicating sufficient evidence to draw a conclusive conclusion. However, the meta-analysis of the low blood pressure risk group and the pneumonia group may have obtained false-positive conclusions, which actually require more trials to confirm the efficacy. The cumulative information did not exceed the anticipated information volume, and neither the SICH group nor the intervention-associated complications group met the conventional threshold or the TSA threshold. More tests are required to determine whether there is a statistically significant difference between the GA group and the CS group (Figure 7).

#### 3.3.3 Subgroup analysis and sensitivity analysis

**Figure 8:**
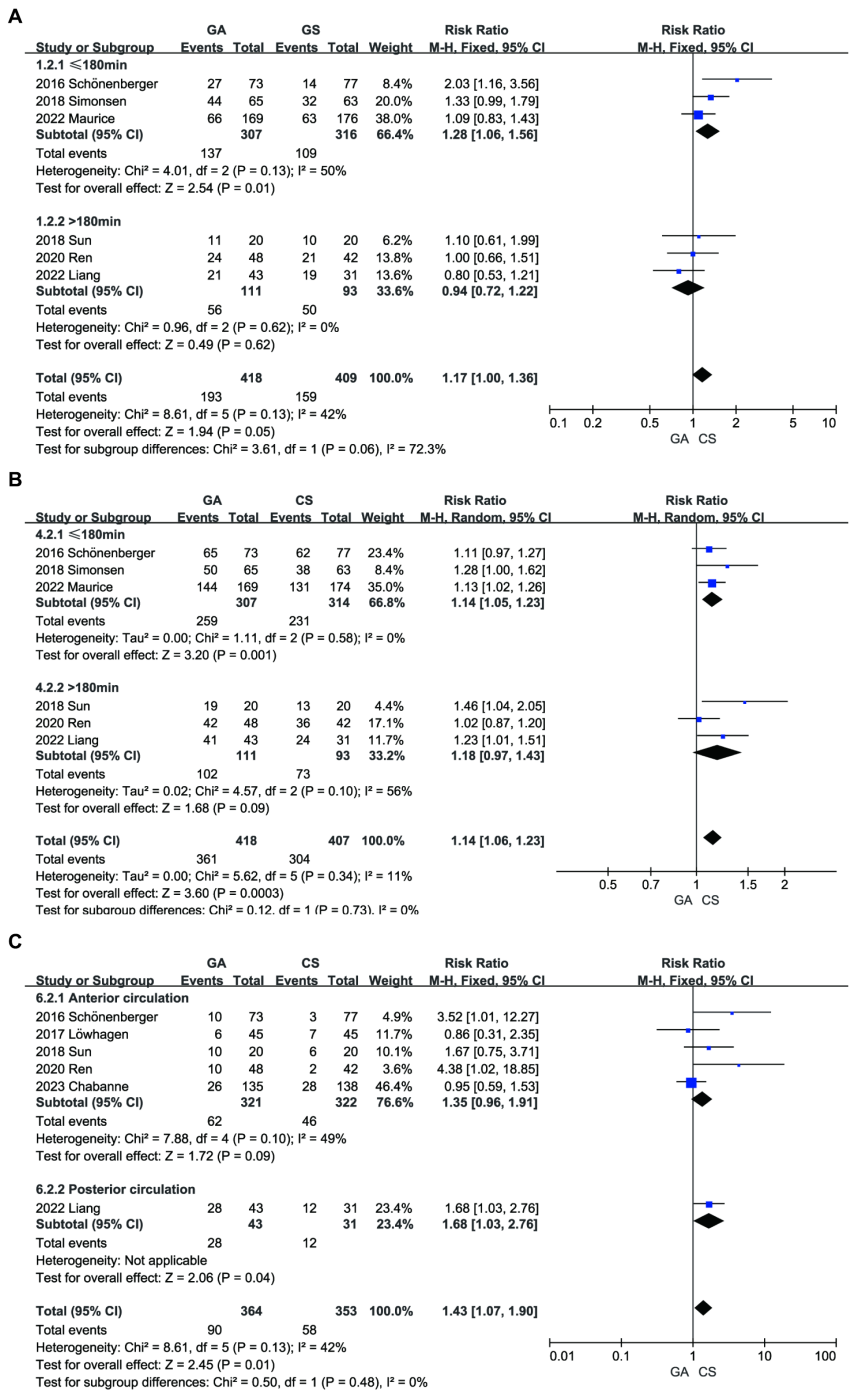
Subgroup analysis. **(A)** The influence of on-site door opening time (time ≤ 180 min or > 180 min) on mRS. **(B)** The influence of on-site door opening time (time ≤ 180 min or > 180 min) on recanalization. **(C)** The influence of subgroup analysis (anterior or posterior circulation infarction) on pneumonia.

We conducted a subgroup analysis based on onset-to-door time and infarction type (anterior or posterior circulation) and found that, under the same conditions of onset and treatment, patients who received treatment within 180 min of onset may achieve better functional independence and reperfusion rates (Figures 8A,B). Liang’s study (13) was the only relevant research on posterior circulation infarction. After excluding this literature and analyzing it again, we found no significant difference in pneumonia incidence between the GA group and the CS group (RR = 1.35, 95% CI: 0.96–1.91, *p* = 0.09) (Figure 8C). Sensitivity analysis was performed by sequentially removing individual studies to evaluate their impact on the combined RR value. The results indicate instability in the sensitivity analysis of the pneumonia group. Upon sequential exclusion of studies by Schönenberger, Ren, and Liang, the aggregated outcomes suggest no significant difference in pneumonia risk between the GA and CS groups (7, 11, 13). Particularly noteworthy is the pronounced reduction in result disparity, especially following the exclusion of Liang’s study (Supplementary Table 1).

We conducted further meta-regression on the included literature, grouping studies based on three criteria [(a) study location in Europe or China; (b) sample size less than or greater than 100 in the experimental and control groups; (c) stroke type classified as anterior or posterior circulation]. However, no evidence was found indicating significant heterogeneity among the results (Supplementary Table 2).

## 4 Discussion

A meta-analysis of eight randomized controlled trials (RCTs) conducted for this study found no evidence of a significant difference between the GA and CS groups in terms of functional independence (an mRS of 0–2) or 90 days mortality when EVT was used for AIS patients. Additionally, the short-term functional evaluation (NIHSS score) showed no obvious difference 24 h following surgery. The GA group had a greater incidence of reperfusion than the CS group, but they also had a higher risk of hypotension and pneumonia. There was no obvious distinction between the two groups in terms of SICH or the effects of the intervention.

Compared with previous meta-analyses reporting higher functional independence of the GA group than the CS group at 90 days, a trend of decreasing differences between the GA group and CS group in the analysis of the final mRS0-2 was observed in the included literature (15–18). Combining with the latest relevant research, we reached a conclusion inconsistent with the previous meta-analyses, that is, the GA group has similar functional independence outcomes to the CS group for AIS patients undergoing EVT (13, 14). The major factors in determining the effectiveness of EVT are successful vascular recanalization and functional independence, and successful recanalization may be closely associated with functional independence 3 months later (25). Due to the better procedural circumstances given by patient immobilization and controlled apnea during GA, the GA group saw a greater rate of recanalization. Additionally, the benefits of GA, such as the monitoring of physiological parameters for oxygenation and hemodynamics, may help improve EVT recanalization rates (26). However, some scholars further proposed that the prognosis evaluation factors of EVT for AIS are more complex, and intraoperative arterial hypotension recurrence is associated with changes in neurologic prognosis after acute ischemic stroke. The patients in the general anesthesia group in the RCTs that were part of our analysis experienced more episodes of hypotension and hypertension despite the fact that both patient groups had standardized hemodynamic control. However, the cumulative duration of hypotension and the outcomes at 3 months were comparable for both groups. During general anesthesia, hyperventilation and hypocapnia may happen. This can lead to cerebral vasoconstriction, which lowers cerebral blood flow and has negative effects on the ischemic penumbra (27). Large fluctuations in blood pressure during anesthesia, combined with comprehensive effects of complications such as general anesthesia tracheal intubation, may be the reason why general anesthesia achieved higher recanalization rates during EVT, and achieved similar outcomes to the CS group for functional independence at 90 days. Therefore, standard circulatory management may play a critical role in reducing adverse outcomes caused by hemodynamic fluctuations.

After performing a subgroup analysis, this study found that patients who were admitted within 180 min of stroke onset in the GA group had higher rates of reperfusion and functional independence compared to the CS group. This is because the shorter time to thrombus formation and vascular occlusion resulted in less neurofunctional damage, which was reflected in better functional recovery after EVT treatment. The latest American Stroke Association (ASA) guidelines recommend limiting the target time from onset to endovascular therapy to within 120 min. The three Chinese studies included in this article all had longer arrival times (more than 180 min), which may result in greater burden for both patients and hospitals (28). The quick identification of patients with probable ischemic stroke and intracranial occlusion and the mobilization of professionals for endovascular intervention are priorities after the beginning of acute stroke. Pre-hospital transportation services are needed for these procedures, and patients’ families, neurologists, nurses, radiologists, interventionalists, and hospital administration departments must all be involved. In combination with the global pandemic during COVID-19, healthcare workers and pre-hospital transportation services require streamlined steps and actions to address challenges (29).

In addition, Liang’s study significantly affected the outcome of pneumonia groups according to subgroup and sensitivity analyses. This is due to the fact that, in contrast to anterior circulation stroke, brainstem involvement predominates in posterior circulation occlusions. Numerous essential physiological processes, including breathing, heart rate, and blood pressure, are controlled by the brainstem. AIS causes directional abnormalities, reduced awareness or coma, and the loss of defensive reflexes by decreasing blood circulation to these vital areas (30). These patients are more likely to experience procedural sedation turning into general anesthesia, have worse general health overall, spend more time unconscious, require tracheal intubation more frequently, and are more likely to develop lung infections. Therefore, it is important to emphasize intraoperative respiratory and circulatory management. Sensitivity analyses suggested instability in the outcome of pneumonia, which may be due to bias from the small sample size. Larger central trials are needed for further identification.

The limitations of the meta-analysis proposed are as follows: (1) only eight studies were included in the meta-analysis, and the sample size was small. Further subgroup analysis is challenging. (2) As most participants in the study were from Europe and China, the results may not accurately reflect their global applicability. (3) Procedural sedation requires an individualized approach, as factors such as the patient’s condition and level of agitation can affect the use of anesthesia drugs. (4) Different anesthesia drugs may have varying effects on outcomes. It may lack universality, particularly requiring high-level interdisciplinary cooperation between neuro-interventionists and anesthesiologists.

## 5 Conclusion

Compared with the CS group, the GA group demonstrated similar functional independence at 90 days after EVT treatment in patients with acute ischemic stroke. The GA group achieved higher rates of reperfusion but also had higher risks of hypotension and pneumonia. The benefits and risks of the GA group compared with the CS group, as confirmed by the TAS analysis, require further validation through additional trials.

## Data availability statement

The original contributions presented in the study are included in the article/Supplementary material, further inquiries can be directed to the corresponding author.

## Author contributions

ZP: Writing – review & editing, Writing – original draft. WL: Writing – original draft, Writing – review & editing. ZY: Conceptualization, Data curation, Formal analysis, Writing – review & editing. HZ: Conceptualization, Data curation, Formal analysis, Funding acquisition, Writing – original draft, Writing – review & editing.
