# Supplementary material for: The effect of general anesthesia and conscious sedation in endovascular thrombectomy for acute ischemic stroke: an updated meta-analysis of randomized controlled trials and trial sequential analysis
Source: Front Neurol. 2023 Dec 8;14:1291211. doi: 10.3389/fneur.2023.1291211 (PMC10740157; doi:10.3389/fneur.2023.1291211)
Supplement: Supplementary file 1 [file Table_1.DOCX]

**Figure S1.** mRS: Summary of outcomes after excluding one study.


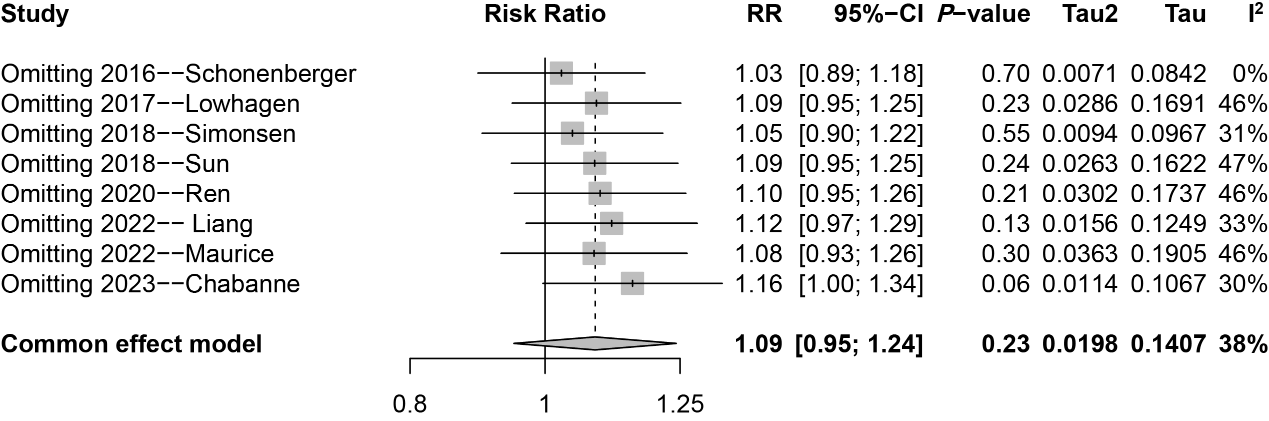


Footnotes: Sensitivity analysis suggests that the outcome is stable.

**Figure S2**. Mortality at 90days: Summary of outcomes after excluding one study.


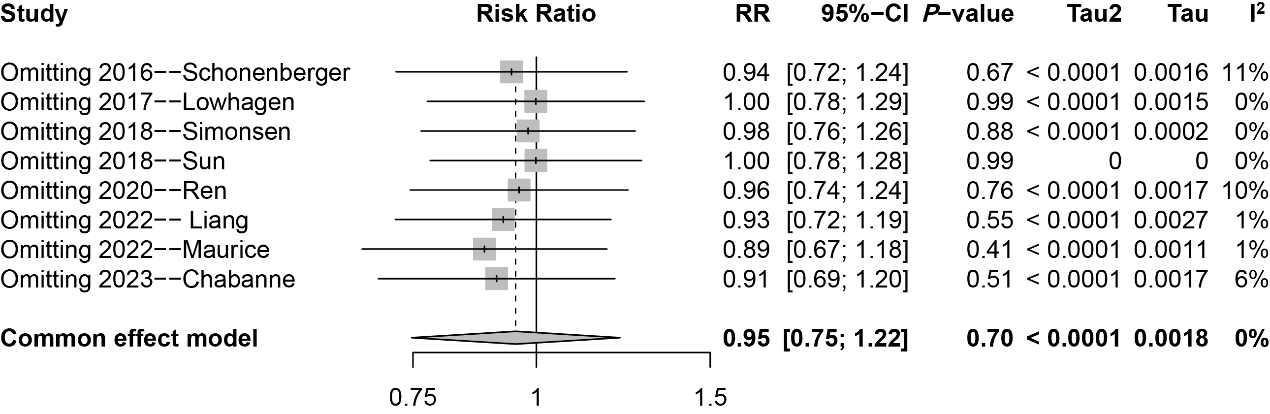


Footnotes: Sensitivity analysis suggests that the outcome is stable.

**Figure S3**. NIHSS score: Summary of outcomes after excluding one study.


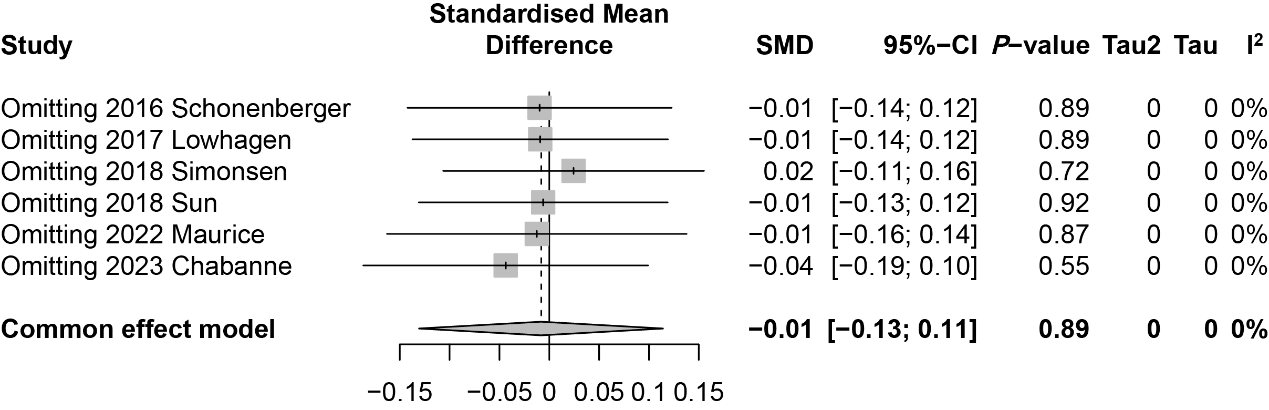


Footnotes: Sensitivity analysis suggests that the outcome is stable.

**Figure S4**. Recanalization: Summary of outcomes after excluding one study.


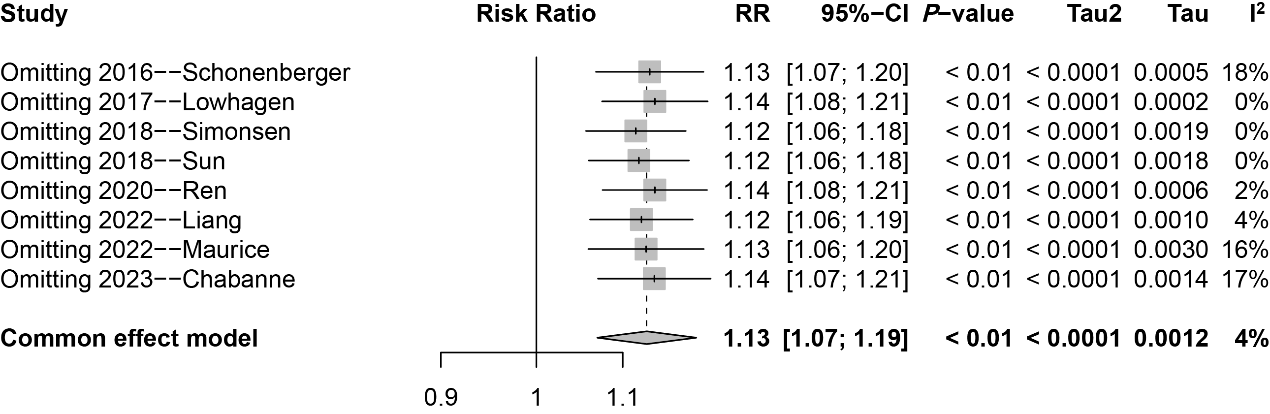


Footnotes: Sensitivity analysis suggests that the outcome is stable.

**Figure S5**. Hypotension: Summary of outcomes after excluding one study.


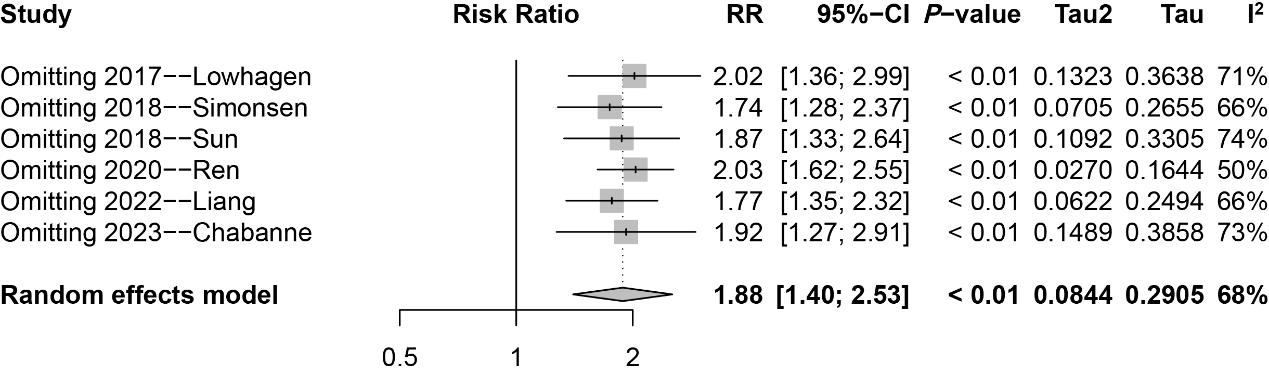


Footnotes: Sensitivity analysis suggests that the outcome is stable. The results suggest partial heterogeneity.

**Figure S6**. Pneumonia: Summary of outcomes after excluding one study.


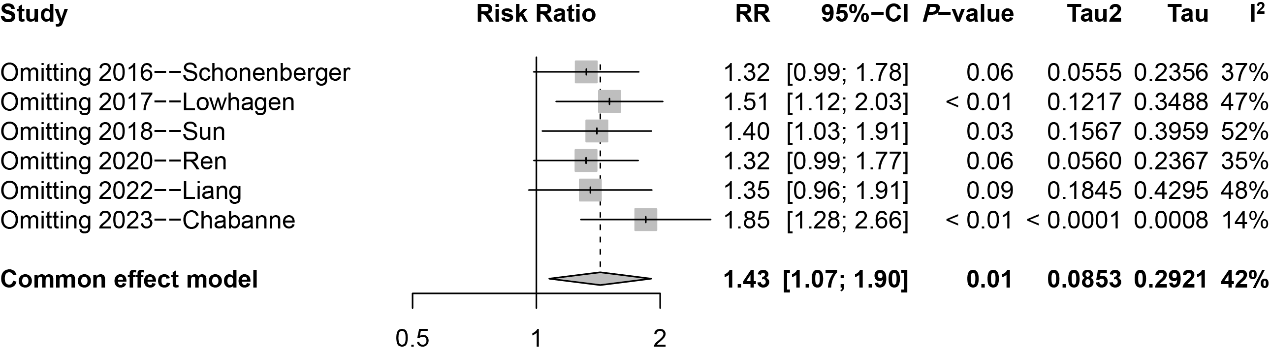


Footnotes: The results indicate instability in the sensitivity analysis of the pneumonia group. Upon sequential exclusion of studies by Schonenberger, Ren, and Liang, the aggregated outcomes suggest no significant difference in pneumonia risk between the GA and CS groups Particularly noteworthy is the pronounced reduction in result disparity, especially following the exclusion of Liang's study.

**Figure S7**. SICH: Summary of outcomes after excluding one study.


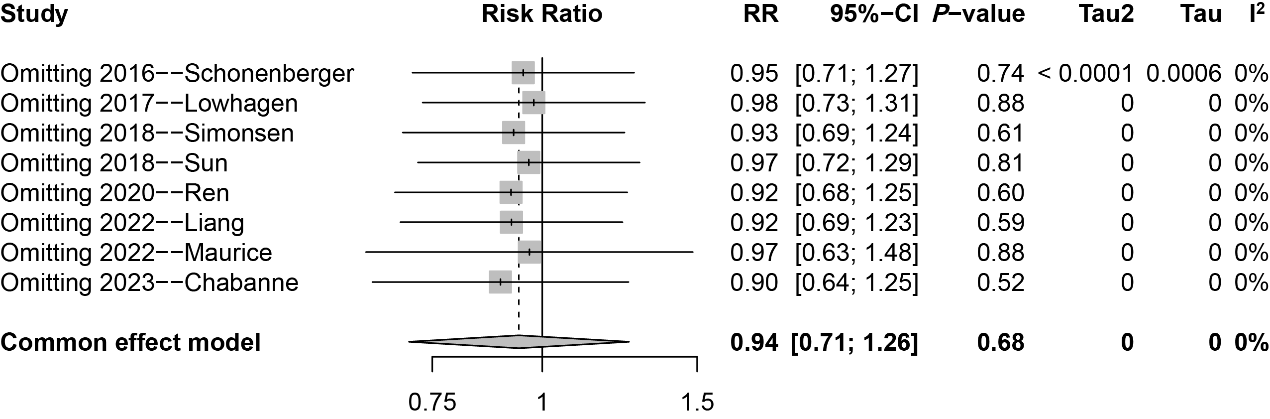


Footnotes: Sensitivity analysis suggests that the outcome is stable.

**Figure S8**. Intervention-associated complications: Summary of outcomes after excluding one study.


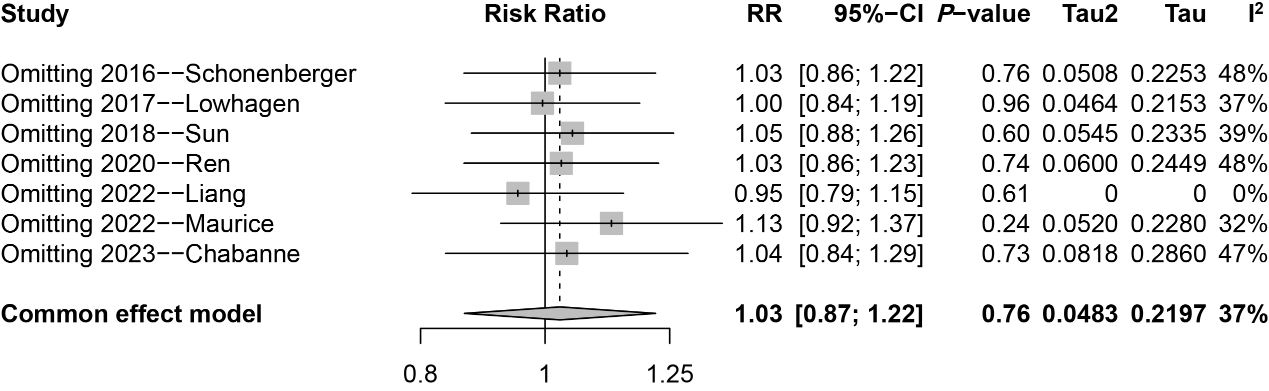


Footnotes: Sensitivity analysis suggests that the outcome is stable.
