# Supplementary material for: The effect of general anesthesia and conscious sedation in endovascular thrombectomy for acute ischemic stroke: an updated meta-analysis of randomized controlled trials and trial sequential analysis
Source: Front Neurol. 2023 Dec 8;14:1291211. doi: 10.3389/fneur.2023.1291211 (PMC10740157; doi:10.3389/fneur.2023.1291211)
Supplement: Supplementary file 2 [file Table_2.DOCX]

**Table S2.1.** Data table for meta regression.

| Year | Study | v1 | v2 | v3 | RR | LCI | UCI |
| --- | --- | --- | --- | --- | --- | --- | --- |
| 2016 | Schonenberger | 1 | 0 | 0 | 2.03 | 1.16 | 3.56 |
| 2017 | Lowhagen | 1 | 0 | 0 | 1.06 | 0.64 | 1.73 |
| 2018 | Simonsen | 1 | 0 | 0 | 1.33 | 0.99 | 1.79 |
| 2018 | Sun | 0 | 0 | 0 | 1.1 | 0.61 | 1.99 |
| 2020 | Ren | 0 | 0 | 0 | 1 | 0.66 | 1.51 |
| 2022 | Liang | 0 | 0 | 1 | 0.8 | 0.53 | 1.21 |
| 2022 | Maurice | 1 | 1 | 0 | 1.09 | 0.83 | 1.43 |
| 2023 | Chabanne | 1 | 1 | 0 | 0.85 | 0.62 | 1.17 |

Footnotes: v1: 0 indicates the study site is in China and 1 indicates it is in Europe; v2: 0 means fewer than 100 people in the test or control group, 1 means more than 100; v3: 0 indicates acute stroke of anterior circulation and 1 indicates acute stroke of posterior circulation; LCI: Lower bound of 95% confidence interval; HCI: Upper bound of 95% confidence interval.

**Table S2.2.** Outcome of meta-regression analysis.

| Subgroups | v1 | v2 | v3 |
| --- | --- | --- | --- |
| R^2^ | 0.00% | 0.00% | 20.33% |
| *P*-value | 0.2762 | 0.3762 | 0.1906 |

Footnotes: There was no evidence of heterogeneity in the groups.
